# The value of a new cardiac magnetic resonance imaging protocol in Myocardial Infarction with Non-obstructive Coronary Arteries (MINOCA) – a case-control study using historical controls from a previous study with similar inclusion criteria

**DOI:** 10.1186/s12872-017-0611-5

**Published:** 2017-07-24

**Authors:** Per Tornvall, E. B. Brolin, K. Caidahl, K. Cederlund, O. Collste, M. Daniel, C. Ekenbäck, J. Jensen, S. Y-Hassan, L. Henareh, C. Hofman-Bang, P. Lyngå, E. Maret, N. Sarkar, J. Spaak, M. Sundqvist, P. Sörensson, M. Ugander, S. Agewall

**Affiliations:** 1 Department of Clinical Science and Education Södersjukhuset, Karolinska Institutet, Stockholm, Sweden; 2 Clinical Science, Intervention and Technology, Karolinska Institutet, Stockholm, Sweden; 3 Molecular Medicine and Surgery, Karolinska Hospital, Stockholm, Sweden; 4 Clinical Sciences Danderyd Hospital, Karolinska Hospital, Stockholm, Sweden; 5 Medicine Huddinge, Karolinska Hospital, Stockholm, Sweden; 6 Medicine Solna, Karolinska Hospital, Stockholm, Sweden; 7 Institute of Clinical Medicine, University of Oslo, Oslo, Norway; 8 Department of Clinical Science and Education Södersjukhuset, Karolinska Institutet, Sjukhusbacken 10, 118 83 Stockholm, Sweden

**Keywords:** Myocardial infarction, Non-obstructive coronaries, Cardiac magnetic imaging

## Abstract

**Background:**

Myocardial Infarction with Non-Obstructive Coronary Arteries (MINOCA) is common with a prevalence of 6% of all patients fulfilling the diagnosis of myocardial infarction. MINOCA should be considered a working diagnosis. Cardiac Magnetic Resonance (CMR) imaging has recently been suggested to be of great value to determine the cause behind MINOCA. The objectives of this paper are to describe the rationale behind the second Stockholm Myocardial Infarction with Normal Coronaries (SMINC-2) study and to discuss the protocol for investigation of MINOCA patients in the light of the recently published position paper from the European Society of Cardiology.

**Methods:**

The SMINC-2 study is an open non-randomised study using historical controls for comparison. The primary aim is to prove that MINOCA patients investigated with the latest CMR imaging technique can achieve a diagnosis in 70% of all cases entirely by imaging. By including 150 patients we will have >80% chance to prove that the diagnostic accuracy can be improved by 20 absolute % with a *p*-value of less than 0.05 when compared with CMR imaging in the SMINC-1 study. Furthermore, in addition to invasive coronary angiography, coronary arteries are evaluated by computed tomography angiography to investigate coronary causes and questionnaires are used to describe Quality-of-Life (QoL). By January 1st 2017, 75 patients have been included.

**Discussion:**

Whether CMR imaging can provide a diagnosis to an adequate proportion of MINOCA patients is unknown. Well-defined inclusion and exclusion criteria will be used to compare a MINOCA cohort from the population with an appropriate control group. Positive results are likely to influence future guidelines of the management of MINOCA. Furthermore, the study will give mechanistic insights into MINOCA in particular in patients with “true” myocardial infarction and describe QoL in this vulnerable group of patients.

**Trial registration:**

Clinical Trials NCT02318498.

## Background

Myocardial Infarction with Non-Obstructive Coronary Arteries (MINOCA) is common with a prevalence of 6% of all patients fulfilling the diagnosis of myocardial infarction [1]. A value of Cardiac Magnetic Resonance (CMR) imaging in MINOCA has recently been suggested. A meta-analysis of five CMR studies of MINOCA showed that one third of the patients had myocarditis and 20% had a “true” myocardial infarction [2]. Today, there are no guidelines on how to investigate and treat MINOCA, however a position paper on MINOCA was recently published by the European Society of Cardiology (ESC) working group on cardiovascular pharmacotherapy [3]. Most importantly, it stated that MINOCA should be considered a “working diagnosis” similar to heart failure. Coronary and non-coronary cardiac mechanisms should be investigated and non-cardiac reasons considered. Great emphasis was placed on CMR imaging in the investigation of MINOCA. CMR imaging was recommended in all MINOCA patients without a clear cause.

The Stockholm Myocardial Infarction with Normal Coronaries (SMINC) study was a study with the primary aim to investigate the role of endothelial function in MINOCA [4]. The results showed that endothelial function and common carotid intima-media thickness were normal when compared to controls. Furthermore, the risk factor profile resembled Coronary Heart Disease (CHD) controls with the exception for a non-atherogenic lipid profile. In the SMINC study, patients with myocarditis were excluded by CMR imaging. Other exclusion criteria were previous myocardial infarction, known cardiomyopathy, severely impaired renal function and Chronic Obstructive Pulmonary Disease (COPD) as well pulmonary embolism and type 2 myocardial infarction. The screening phase of the SMINC study of all patients investigated with CMR imaging showed that it was possible to assign a definitive diagnosis to 50% of the patients by performing CMR imaging and adding clinical information in particularly on the occurrence of Takotsubo stress CardioMyopathy (TCM) [5]. Major limitations of the CMR imaging in the SMINC study were that it was performed in median 12 days after the acute event and that the latest CMR imaging technique including T1 mapping to detect oedema was not performed. Furthermore, at that time there was no clear CMR imaging-based diagnosis for TCM. The second SMINC study (SMINC-2) is on-going with the *primary aim* to give 70% of the patients a definite diagnosis purely on the basis of the CMR imaging results. To be able to do this, the protocol states that CMR imaging should be performed 2-4 days after hospitalisation because of MINOCA and to include T1 mapping in addition to T2 oedema sequences. An overall *secondary aim* is to test a protocol on how to investigate MINOCA patients including further characterisation of these patients with emphasis on coronary imaging and stress. The objectives of this paper are to describe the rationale behind the SMINC-2 study and to discuss the protocol of investigation of MINOCA patients in the light of the recently published position paper [3].

## Methods/design

The study design is an open non-randomised multi-center study using historical controls from the SMINC study for comparison. The primary aim is to prove that patients investigated with CMR imaging can achieve a definite diagnosis in 70% of all MINOCA cases entirely by imaging (Clinical Trials NCT02318498). By including 150 patients, we will have >80% chance to prove that the diagnostic accuracy can be improved by 20 absolute % with a *p*-value of less than 0.05 when compared with our previous sample [5]. By January 1st 2017, 75 patients have been included. The study is supported by grants provided by the Stockholm County Council (ALF project) and Swedish Research Council.

### Inclusion criteria

Consecutive patients from the five major hospitals in Stockholm, 35-69 years old, fulfilling the existing diagnostic criteria of myocardial infarction [6] with angiographically normal coronary arteries will be included. Coronary angiography will be performed according to the individual laboratory including 4-5 projections of the left coronary artery and 2-3 projections of the right coronary artery. Patients with angiographically completely normal coronary angiography or with signs of diffuse coronary atherosclerosis with no lesion giving rise to a measurable stenosis of any percentage will be included. If doubt a Quantitative Coronary Analysis (QCA) should be performed on the projection showing the most narrow intrusion and the patient excluded if the results shows a stenosis of >20% that is the lowest limit of detection with QCA. All coronary angiograms should be evaluated by two experienced angiographers.

### Exclusion criteria

Patients without sinus rhythm on admission ECG, pulmonary embolism, COPD, previous myocardial infarction, known cardiomyopathy or severe renal impairment (serum creatinine above 150 umol/l) will be excluded.

Patients with a pacemaker and claustrophobia will be excluded due to interference with interpretation or possibility to perform CMR imaging.

### Investigations

**Fig. 1:**
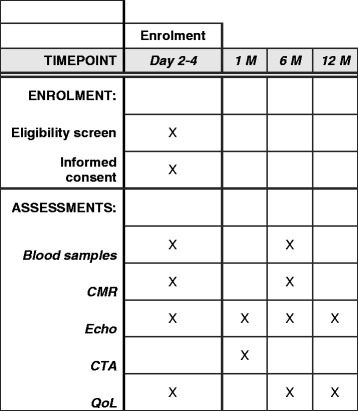
SPIRIT figure of the Stockholm Myocardial Infarction with Normal Coronaries study 2. CMR=Cardiac magnetic resonance imaging; CTA = Computed Tomography Angiography; Echo = Echocardiography; QoL = Quality-of-Life

An overview including the time-frame of investigations is shown in the Fig. 1.

CMR is performed by a Siemens Aera 1,5 T® including the following steps: 1. Scout through thorax with transversal, coronar and sagital stacks with trueFISP-sequences, without ECG triggering. 2. Vertical long-axis through left ventricle with trueFISP without ECG triggering. 3. Horisontal long-axis through left ventricle with trueFISP cine, with ECG triggering. 4. Pre-contrast T1-mapping with MOLLI (Modified Look-Locker Inversion recovery) sequence containing several ECG-triggered single shot trueFISP in end-diastole with different times of inversion. Sequences should be sampled in short-axis, 2,3 and 4 chamber view stacks. 5. Pulmonary transition time, saturation recovery trueFISP perfusion, single slice short-axis through left atrium, 20 mm slice thickness, without ECG triggering, parallel -imagination with GRAPPA factor 4 with 2 ml bolus of contrast (Dotarem®) + 20 ml NaCl, 4 ml/s injected by automated injector. 6. Further contrast injection (Dotarem®) to double dose injected by automated injector. 7. Cine trueFISP in short-axis, 2,3 and 4 chamber view stacks to study left ventricular function. 8. TI-scout followed by phase sensitive inversion recovery (PSIR) late gadolinium enhancement (LGE) in short-axis, 2,3 and 4 chamber view stacks, to study infarction and microvascular obstruction. Should be made at least 10 min after contrast injection. 9. MOLLI post-contrast T1-mapping in short-axis, 2,3 and 4 chamber view stacks*.* 10. Phase contrast-based flow measurement sequence, without breath-holding, through aorta ascendens and pulmonary artery.

*Acute myocardial infarction* is defined as localized subendocardial late gadolinium enhancement (LGE) with corresponding oedema (T1 mapping).

*Acute myocarditis* is defined as diffuse mainly subepicardial LGE with corresponding oedema (T1 mapping).

*Acute TCM* is defined as presence of myocardial oedema (T1 mapping) and/or transient wall motion abnormalities with circumferential distribution with absence of corresponding LGE and coronary artery distribution.

Computed tomography angiography (CTA) is performed using a 64-slice computed tomography scanner with ECG-triggered, prospective technique and with parameter settings resulting in submillimeter isotropic voxels. Patients with a heart rate > 65 beats/min will be given betablockers, orally or intravenously, before the scan. Patients with a systolic blood pressure > 100 mmHg will be given sublingual nitroglycerine before the scan. First, a non-enhanced scan will be performed enabling measurement of calciumscore. Second, nonionic iodinated contrast agent will be administered as a standard bolus volume but with individually measured time of delay. Data within the end-diastolic phase will be reconstructed in 5% steps and thereafter analyzed using dedicated software with post-processing tools such as multiplanar reformation, curved multiplanar reformation and maximum intensity projection. Images will be analyzed semi-quantitatively regarding number, location, severity and quality of coronary plaques by 2 observers who are blinded to the clinical information where disagreement will be solved by consensus.

Echocardiography is performed on GE Vivid E9® scanners. All registrations besides CW/PW Doppler consist of three cardiac cycles. The ejection fraction will be calculated using Simpson’s rule. Regional wall motion will be classified by visual inspection of wall thickening, classifying segments with less than 30% thickening as hypokinetic, less than 10% as akinetic, and segments with systolic thinning or eccentric excursion as dyskinetic. 2D loops of from the three apical views are analyzed using the longitudinal 2D strain Q-analysis module in EchoPAC BT11.2® (GE, Horten, Norway). After marking the myocardial borders as the region of interest (ROI), the application semi-automatically generates strain curves and peak systolic strains, and also automatically calculates the proportion of shortening that takes place after aortic valve closure (post systolic index). Furthermore the temporal derivation to strain rate can be performed. The software calculates global averages as well as segmental values for all variables, and both will be recorded. Color coded tissue Doppler loops from the same apical views will be analyzed in the TDI Q-analysis module. ROIs will be placed in the two basal segments in each view, in order to measure the peak systolic and early diastolic velocities, as well as the timing of the events of the cardiac cycle, which will be used to calculate the myocardial performance index for the left ventricle, and in the same manner for the right ventricle. The standard diastolic parameters of mitral inflow velocities, E/E’, and pulmonary venous flow will be measured. The E waves acquired during free breathing and after the Valsalva maneuver will be analyzed in a custom LabVIEW® (National Instruments, Austin, USA) interface where, by fitting the curve generated by the equation for a simple harmonic oscillator to the maximum velocity envelope of each E wave, the equation constants *k*, *c*, and *x* are calculated. These constants correlate closely to chamber stiffness, viscoelastic energy loss and load, respectively. Furthermore, the results are used to calculate the time constant of isovolumic chamber relaxation (*tau*), the stiffness and relaxation components of the deceleration time of the E wave, a load independent index of diastolic filling and stiffness.

Laboratory investigations are performed by routine clinical chemistry. Haematology, electrolytes, cystatin C, glucose, lipids and NT-proBNP, thyreoid function tests, hsTroponinT, D-dimer, metoxycatecholamines and urine albumin/creatinine ratio, oral glucose tolerance test (75 g with measurement of glucose after 2 h), HbA1C and cortisol in saliva (morning and evening). All patients will be investigated haematologically to exclude proneness to thromboembolism and systemic lupus erytematosus at 6 months according to the routine of Karolinska University Laboratory.

Quality-of-Life is investigated: Rand 36 (former SF-36), Multidimensional Fatigue Inventory (MFI-20), Perceived Stress Scale (PSS-14) and Hospital and Anxiety Depression Scale (HADS).

## Discussion

The primary objective of the present paper is to describe the rationale behind the SMINC-2 study that has the aim to test if early CMR imaging using the latest technique can give more than 70% of all MINOCA patients a CMR imaging-based diagnosis. The main diagnoses that are possible to acquire are myocarditis, TCM and “true” myocardial infarction. Our previous meta-analysis of CMR imaging findings in MINOCA showed that one third of the patients had myocarditis and 20% had “true” myocardial infarction [2, 8]. These new studies shows that early CMR imaging (median 3, respectively 6 days) with conventional T2 oedema sequences gave the patients a definite diagnosis in 90%, respectively 87% of the cases. The reasons for these extraordinary figures are possibly due to study design where the results by Emrich and coworkers [7] are limited due to the retrospective design possibly including patients with already known or suspected diagnosis whereas in the prospective study by Pathik and co-workers [8] all-comers were included without exclusion criteria. Both studies were able to diagnose TCM by CMR imaging (10%, respectively 27%) thereby improving the diagnostic performance of CMR imaging. The results of the meta-analysis and the recent studies [2, 7, 8] support the idea that early CMR imaging with the latest CMR imaging technique can give the diagnosis in the vast majority of MINOCA patients thus being an important diagnostic tool as pointed out in the recent position paper by the ESC [3].

A secondary objective of the present paper is to discuss the protocol of investigation of MINOCA patients in the light of the recently published position paper by the ESC [3]. In the position paper, a left ventriculogram or echocardiography is recommended if MINOCA is found during coronary angiography. This is recommended routinely by our local guidelines and will therefore be performed in the present study. Further invasive studies, suggested in the position paper, to exclude ruptured plaques, coronary dissections, microvascular dysfunction and coronary spasm are not routinely performed. The value of these investigations has to be proven before they can be recommended. Instead, in the present study a non-invasive CTA is performed 1 month after the acute event. The timing was made to decrease the risk for contrast-induced nephropathy after investigations with coronary angiography, including left ventriculography, and CMR. This investigation has the advantage that it can detect plaques not visible on invasive coronary angiography. It can also to some degree determine if the plaque is stable or unstable [9]. CTA will make it possible to investigate the cause of “true” myocardial infarction by studying myocardial infarction determined by CMR imaging in relation to supplying coronary artery. Furthermore, CTA is an accurate method to exclude coronary dissections [10]. Unfortunately, there are no reliable non-invasive examinations to exclude microvascular dysfunction and coronary spasm. Other investigations that are performed in SMINC-2 suggested by the position paper [3] include sampling of catecholamines to exclude pheocromocytoma, D-dimer to exclude pulmonary embolism and tests to exclude inherited causes of thromboembolism. The value of the results of all these investigations will be determined at the end of the study and the results will most likely support or refute the algorithm suggested by the recent position paper.

### Strength and limitations

The major strengths of SMINC-2 are the fairly high number of patients included from several hospitals being representative for a north European population and in comparison with several previous studies it is prospective and the first one with a control group. Limitations include the fairly strict age criteria and that it will not include patients with near significant coronary artery stenosis to be able to have the SMINC study as a control group. The reasons for the strict age criteria in the SMINC study were that it was unlikely that all patients would receive a coronary angiography if patients were below 35 and above 69 years of age. The reason for the lower age limit is that the likelihood is much higher for myocarditis than CHD and thus the diagnosis would be made without a coronary angiography. Regarding the upper age limit, many patients 70 years and older with suspected CHD were treated conservatively at the time of the start of the SMINC study 10 years ago. This together with the fairly strict inclusion and exclusion criteria may somewhat limit the representativity of the study.

## Conclusion

Whether CMR imaging can provide a diagnosis to an adequate proportion of MINOCA patients is unknown. Well-defined inclusion and exclusion criteria will be used to compare a MINOCA cohort from the population with an appropriate control group. Positive results are likely to influence future guidelines of the management of MINOCA. Furthermore, the study will give mechanistic insights into MINOCA in particular in patients with “true” myocardial infarction and describe QoL in this vulnerable group of patients.
